# Comparison of the clinical effect between the lower sternal incision and the left parasternal fourth intercostal incision in the transthoracic closure of ventricular septal defect

**DOI:** 10.1186/s13019-021-01543-6

**Published:** 2021-06-07

**Authors:** Jun Ma, Wenlin Shangguan, Liang-Wan Chen, Dong-shan Liao

**Affiliations:** 1 Affiliated Hospital of Jining Medical University, Jining, China; 2 Department of Cardiovascular Surgery, Union Hospital, Fujian Medical University, Fuzhou, 350001 China; 3 Key Laboratory of Cardio-Thoracic Surgery, Fujian Medical University), Fujian Province University, Fuzhou, China

**Keywords:** Congenital heart diseases, Ventricular septal defect, Cardiac intervention

## Abstract

**Background:**

To analyze the clinical effect of two different ways of minimally invasive transthoracic closure in children with ventricular septal defect (VSD).

**Methods:**

From January 2015 to July 2019, 294 children with VSD were enrolled in the Fujian Medical University Union Hospital. Patients were divided into two groups – those who underwent VSD closure through the left sternal fourth intercostal incision (group A: *n* = 95) and the lower sternal incision (group B: *n* = 129).

**Results:**

The operation time, bleeding volume, postoperative mechanical ventilation time, postoperative intensive care unit (ICU) monitoring time, postoperative hospitalization time and complication rate in group A were significantly lower than those in group B (*P* < 0.05). There was no significant difference between the two groups in the operation success rate, mechanical ventilation time and total hospitalization cost (*P* > 0.05).

**Conclusion:**

The transthoracic closure of ventricular septal defect through the left sternal fourth intercostal incision is feasible, safe, cosmetic, and worth popularizing.

## Background

VSD is one of the most common congenital heart diseases (CHD), accounting for approximately 20% of all CHD, of which 80% is perimembranous ventricular septal defect (PmVSD) [1]. There are many procedures to treat VSD, such as traditional repair of ventricular septal defect (RVSD), transthoracic closure of ventricular septal defect (TTCVSD), percutaneous closure of ventricular septal defect (PTCVSD). This TTCVSD procedure will only leave a small incision on the skin surface with impressive cosmetic effect [2]. The purpose of this study is to compare the advantages and disadvantages of the two surgical approaches of the left sternum intercostal incision and the substernal incision to provide more evidence for the rational selection of VSD operation.

## Data and methods

From January 2015 to July 2018, 224 children with VSD, 103 males and 121 females, aged from 4 to 36 months and weighing 6-16 kg, were selected to be treated by transthoracic closure of VSD in Fujian Medical University Union Hospital. They were divided into two groups by types of surgical procedure: group A (the left parasternal fourth intercostal incision) 95 cases, group B (the lower sternal incision) 129 cases. The selected indicators were the operative time, bleeding volume, postoperative mechanical ventilation time, ICU duration, hospital stay, total hospitalization cost, surgical success rate and incidence of various complications. Data were obtained from electrocardiogram, echocardiography, operative data and postoperative follow-up data of the two groups.

### Inclusion criteria

① transesophageal echocardiography (TEE) measurement: 3 mm ≤ VSD diameter ≤ 10 mm; ② age ≤ 3 years old, or weight ≤ 10 kg; ③ weight > 10 kg, but the TVCVSD operation failed or patients refused TVCVSD; ④ without VSD right to left shunt;; ⑤ the distance from the upper edge of VSD to the right coronary valve of aorta ≥1 mm, and the distance from the lower edge of VSD to the apex ≥3 mm.

### Exclusion criteria

① TEE measurement: 10 mm < VSD diameter or VSD diameter < 3 mm; ② septicemia; ③other complex cardiac malformations requiring simultaneous correction.

Instruments and equipment: Philips ie33 color Doppler ultrasound instrument with children’s esophageal ultrasound probe S7-3T was chosen and the probe frequency was 3 to 5 MHz. The occluders (symmetrical and eccentric) was manufactured by; and other surgical objects. Any contraindications of esophageal ultrasound examination, such as esophageal stenosis or esophageal surgery, should be avoided [3].

### Operation steps

The patients were under the condition of general anesthesia, tracheal intubation and in supine position. The esophageal ultrasonic probe was placed to measure the diameter of VSD. The appropriate occluder was selected according to the diameter and location of VSD.

**Fig. 1:**
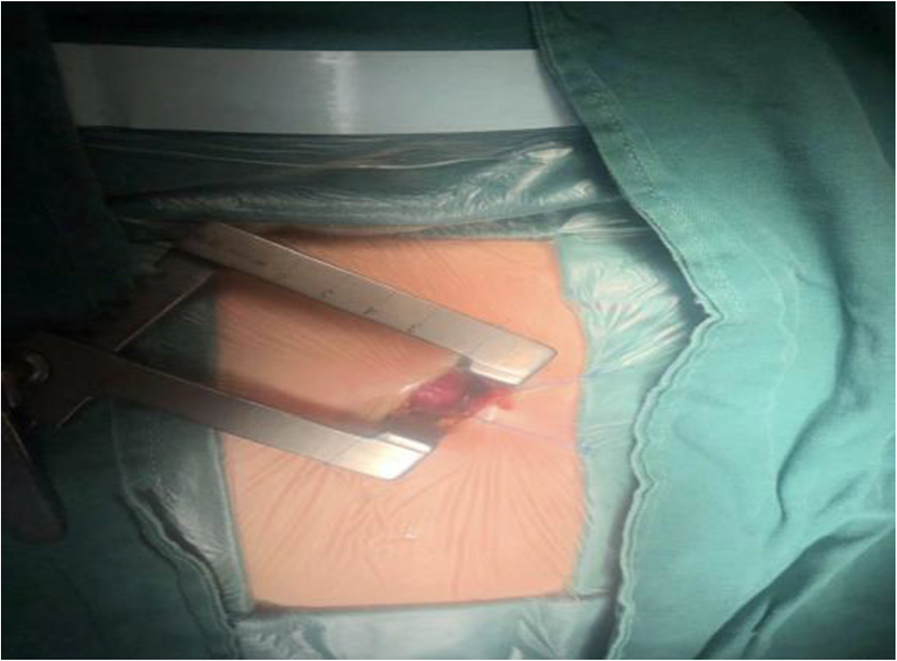
left sternal fourth intercostal incision

Group A: A small oblique incision between the 4th rib on the left edge of the sternum was performed to expose the right ventricle (as shown in Fig. 1).

**Fig. 2:**
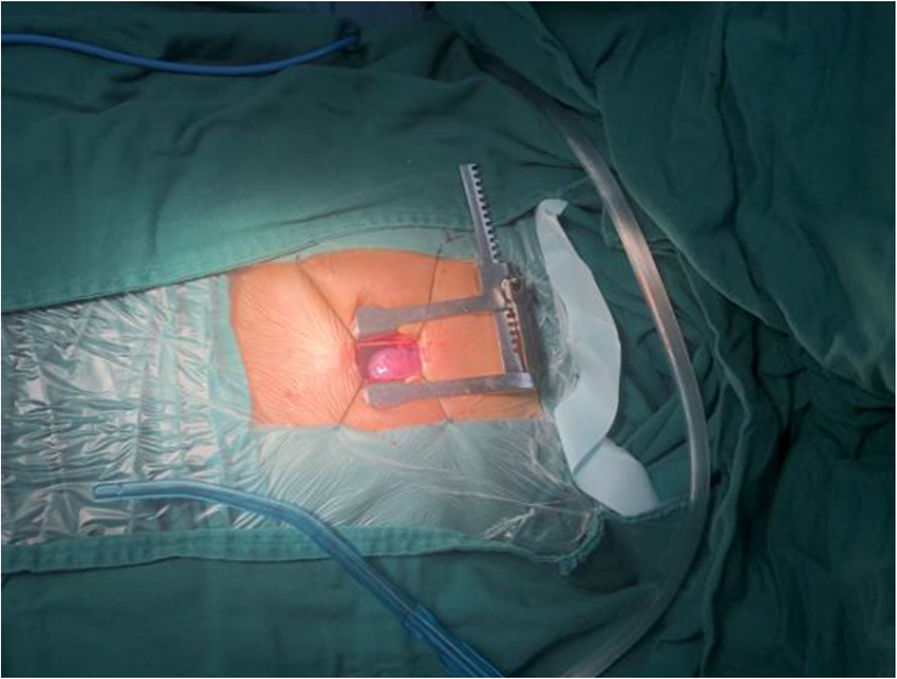
lower sternal incision

Group B: A small incision about 3 to 5 cm at the lower sternum was performed (as shown in Fig. 2).

**Fig. 3:**
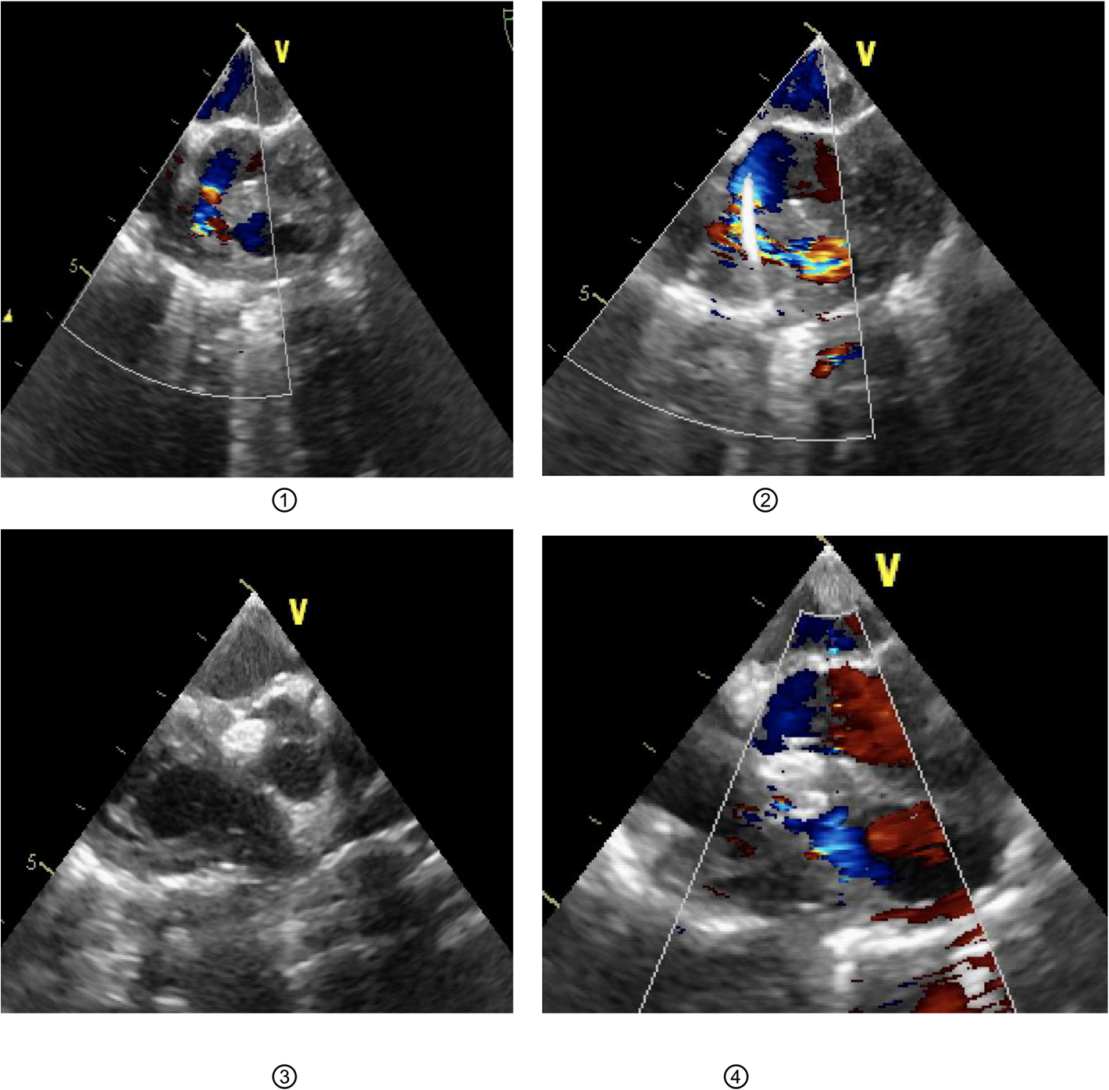
Blocking operation diagram under TEE. ①:Evaluate the VSD size under TEE and select the appropriate occluder.②:The guide wire passed through the ventricular septal defect through the trocar guided by ultrasound.③:Release the left ventricle occlusion umbrella and pull it close to the left ventricle④:There was no significant residual shunt in the TEE assessment

The location shortest distance to VSD on the right ventricular outflow tract was determined by TEE. A purse string was sutured by 5–0 prolene suture and then heparinization was taken at the dosage of 1 mg / kg heparin. After the puncture needle penetrated the right ventricular wall through the center of the purse string, the needle core was drawn out. The guide wire passed through the ventricular septal defect through the trocar guided by ultrasound, afterwards the delivery track would be established. Then the delivery sheath followed the guide wire and passed the defect until it reached the left ventricle. Under the guidance of ultrasound, the steel wire with the occluding umbrella was pushed into the left ventricle and secured,the left ventricular surface of the occluder was then released. Afterwards the pushing wire, the delivery sheath and the occluder was pulled back at the same time, so that the left ventricular surface of the occluder was close to the left ventricular surface of VSD. Meanwhile, the fluctuation of blood pressure and heart rate should carefully monitored during the pulling back. The delivery sheath could only be withdrawn and then the right ventricular surface was released if no aortic regurgitation or tricuspid dysfunction was detected. The firmness of occluder placement was examined by pushing and pulling the pushing wire. If so and there was no residual shunt, no valve dysfunction, the steel wire and the occluder would be separated. Then the delivery sheath and steel wire could be withdrawn. The purse could be knotted and finally protamine could be given. (as shown in Fig. 3).

Group A: The drainage tube was placed in the pericardial cavity.. Group B: The drainage tube was placed in the pericardial cavity.

Postoperative management: The patient was well attended until emergence. Oral aspirin was given 3 to 5 mg / (kg · d) for 3 months. The chest X-ray, electrocardiogram and echocardiography were reviewed at 3 to 5 days after the operation.

Comparative index: The chest ratio was measured by chest X-ray. Diameter and type of VSD, pulmonary artery pressure was measured by TEE. The operation time was recorded from the beginning of skin incision to the end of skin sewing. Bleeding volume referred to the sum of intraoperative bleeding volume and postoperative drainage volume. The indications of successful operation are no residual shunt, no valve dysfunction and no arrhythmia. Only the children with successful operation were counted.

Statistical processing: Statistical software with SPSS 24.0 was used for analysis. T test was used to analyze the data, which was expressed in mean ± standard deviation ($\overline{x}$ ±s). Analysis of variance was used to compare the mean between groups. The counting data were expressed in frequency and percentage (n, %), and chi-square test was used for comparison between groups.

## Results

Both groups of patients had satisfactory outcomes for VSD closure. As shown in Table 1 There were no differences in preoperative between the two groups. Table 2 shows that group A had significantly shorter operation time, postoperative drainage volume, estimated intensive care time and hospitalization time. However, there were no significant differences in the operation success rate, the duration of mechanical ventilation and total cost of hospitalization between group A and group B. (*P* > 0.05) Table 3 shows that the postoperative complications occluder shift, residual shunt, aortic insufficiency, cardiac insufficiency, arrhythmia, pulmonary infection, pneumothorax, pleural effusion, incision infection / poor healing and hemolysis were of no significant differences (*p* > 0.05) between the two groups.

**Table 1:** Preoperative data comparison between two groups of patients

Item	Group A(*n* = 95)	Group B(*n* = 129)	*P*
**Gender (M/F)**	45/50	58/71	/
**Age (year)**	1.33 ± 0.62	1.31 ± 0.76	> 0.05
**Weight (kg)**	9.73 ± 2.42	9.67 ± 2.53	> 0.05
**Cardiothoracic ratio**	0.58 ± 0.28	0.57 ± 0.28	> 0.05
**Size of VSD (mm)**	4.90 ± 3.05	4.40 ± 3.08	> 0.05
**Types of VSD (TTE)**			
Subpulmonic VSD(n, %)	14(14.73%)	18(13.95%)	> 0.05
Permembranous VSD(n, %)	68(71.6%)	90(69.77%)	> 0.05
Muscular VSD(n, %)	13(13.68%)	21(16.28%)	> 0.05
**Pulmonary arterial pressure grading (TTE)**			
Nomal(n, %)	78(82.11%)	102(79.07%)	> 0.05
Mild(n, %)	10(10.53%)	15(11.63%)	> 0.05
Moderate(n, %)	5(5.26%)	8(6.20%)	> 0.05
Severe(n, %)	2(2.10%)	4(3.10%)	> 0.05
**Pulmonary infection(n, %)**	10(10.53%)	15(11.63%)	> 0.05
**Cardiac insufficiency(n, %)**	9(9.47%)	13(10.07%)	> 0.05
**Period of follow-up (month)**	12	12	/

*Abbreviation*: *TTE* transthoracic echocardiography

**Table 2:** Comparison of intraoperative and early postoperative data between the two groups

Item	Group A	Group B	*P*
Operative time (min)	35.67 ± 7.01	63.38 ± 6.53	< 0.001
Bleeding volume (ml)	2.93 ± 11.78	62.59 ± 38.92	< 0.001
Mechanical ventilation time(h)	2.58 ± 0.53	2.63 ± 0.56	> 0.05
Intensive care unit time(d)	0.90 ± 0.27	1.05 ± 0.26	< 0.001
Postoperative hospital stay(d)	4.14 ± 2.28	7.07 ± 3.55	< 0.001
Hospital costs (RMB)	35,116.46 ± 2462.90	39,278.33 ± 3402.32	> 0.05
Operation success rate(%)	87 (91.58%)	119 (92.25%)	> 0.05

*Abbreviation*: *RMB* renminbi

**Table 3:** Comparison of postoperative complications between the two groups

Item(n, %)	Group A	Group B	***P***
Occluder shift	2, 2.30%	3, 2.52%	> 0.05
Small residual shunt	4, 4.60%	4, 3.36%	> 0.05
Aortic insufficiency	2, 2.30%	3, 2.52%	> 0.05
Cardiac insufficiency	1, 1.15%	1, 0.84%	> 0.05
Transient arrhythmia	7, 8.05%	9, 7.56%	> 0.05
Pulmonary infection	7, 8.05%	10, 8.40%	> 0.05
Pneumothorax	0, 0	3, 2.52%	> 0.05
Pleural effusion	0, 0	2, 1.68%	> 0.05
Incision infection / poor healing	0, 0	3, 2.52%	> 0.05
Hemolysis	1, 1.15%	2, 1.68%	> 0.05
Anemia	4, 4.60%	23, 19.32%	< 0.05
Malformed chest	0, 0	6, 5.04%	< 0.05

Patients were followed up until 1 year after the operation. All patients received electrocardiogram, TTE and physical examination at 3 months and 1 year after surgery. During the follow-up period, no serious complications or deaths were observed in all patients.

## Discussion

VSD is one of the most common congenital heart diseases, of which PmVSD accounts for the most. The surgical intervention brough huge clinical benefits to patients and the improvement of the procedure is still expected by many. With the progression of modern surgical technology, not only the health benefit is being persued, but also the cosmetic improvement. The absolute minimalization of procedural harm and cost has become the common pursuit of doctors and patients. As of now, doctors and Patient’s parents tend to choose minimally invasive surgery if possible [4].

There is no doubt that surgical repair has better extensive indications and high success rate [5, 6]. Compared with traditional open chest repair, the most obvious advantage of minimally invasive transcatheter closure is the avoidance of Cardiopulmonary bypass (CPB), CPB-related complications, especially those related to brain damage, which have been widely reported [7, 8]; and the incidence of arrhythmia after minimally invasive transcatheter closure is lower than the former [9–11]. There are two kinds of minimally invasive closure of VSD, TTCVSD and PTCVSD. PTCVSD has many disadvantages, such as complicated operation, radiation damage to doctors and patients, allergy and renal function damage caused by the contrast medium, etc., and it has many limitations, so it is necessary to carefully monitor patients [12].

Ultrasound-guided TTCVSD is a new minimally invasive cardiac surgical procedure in recent years [13–15] This type of operation has higher requirements on the surgeons and the ultrasound doctors. It requires the surgeon to be familiar with the basic knowledge of cardiac anatomy and color Doppler ultrasound, and the ultrasound doctor to be familiar with all facets. This needs the coordination and cooperation of the surgical department, ultrasound department and anesthesia department and other teams [16].

The success rate is what the operator should focus on at first. Our hospital has been performing TTCVSD for a long time and has extensive experience [17]. Sixty-one adult patients underwent transthoracic device closure of pmVSD with the lower sternal incision [18]. All the procedure were successful with little complications. The success rate of TTCVSD with lower sternal incision was the same even compared with surgical repair with right infra-axillary thoracotomy or with right submammary thoracotomy [19].

There is no significant statistical difference in the preoperative data between the two groups of patients. The choice of the TTCVSD route mainly respects the patient’s parents. Though the two routes of TTCVSD can get the same success rate, the differences between them are significant so much.

The shorter the operation time is, the shorter the anesthesia time is, the more beneficial it is for the postoperative recovery of the children. In this study, group A did not need the sternotomy, so the operation time of group A is much less than that of group B (*P* < 0.001), the amount of postoperative drainage fluid in group A was much less than that in group B (*P <* 0.001), and the incidence of postoperative anemia in group A was lower than that in group B (*P* < 0.05). This is partly because the fourth intercostal incision on the left margin of the sternum avoids the internal thoracic contour artery. When device closure failed, the lower sternal incision can enlarge the incision and transform the surgical repair; the left parasternal fourth intercostal incision needs to choose the middle open chest repair, which cause another wound, but before the operation, we will evaluate the feasibility of the operation through TEE to improve the success rate of the operation, so as to reduce the possibility of one more wound.

The incidence and severity of postoperative complications are important indicators for judging the safety of surgical methods. Transcatheter device closure of VSD has been widely used in clinical practice [20, 21] In this study, although the surgical results in both groups were satisfactory and the surgical trauma was minimally invasive, it is inevitable that there will be postoperative complications in both. First of all, arrhythmia is the most serious complication. The incidence of arrhythmia in both groups is acceptable and there is no significant difference. In the two groups some patients were suffered from arrhythmia (atrial/ventricular premature beats, and right/left bundle branch block), which were transient and were easy to treat with drugs or by spontaneous recovery. Aortic insufficiency is another complication of interventional treatment of ventricular septal defect. Which was mainly found in the subpulmonic VSD. Although we chose to use an eccentric occluder, it still occurred. There was no significant difference in aortic valve reflux between the two groups [10, 15, 22].

There were significant differences in sternal deformities between the two groups. The reason may be that in group A, the sternum was not damaged during the operation, and the incidence of postoperative thoracic deformity was 0. In group B, the lower sternal segment was damaged, It may also be due to the small sample size in this report which might present a biased high rate of sternum deformity. The wound of group A was close to the left edge of the sternum, thus greatly reduces breast injury. The wound of group A injured the intercostal muscles. There is a possibility of crowding of ribs in follow up. This may require a longer follow-up to confirm it. In short, from a cosmetic point of view, the left parasternal fourth intercostal incision is more attractive than the lower sternal incision.

This study has guiding significance for the reasonable selection of surgical methods for children with VSD, especially for children younger than 3 years old or weighing less than 10 kg who have failed percutaneous VSD occlusion. The limitation of this study is that this study is a retrospective rather than a prospective comparative study. There is no randomized and balanced grouping. The follow-up time is short. The data comes from a single clinical center. It needs to accumulate more cases and follow up for a longer time to observation and summary.

## Conclusions

Ventricular septal defect occlusion through the lower sternal incision and the left parasternal fourth intercostal incision is a feasible, minimally invasive and safe approach. Ventricular septal defect occlusion through the left parasternal fourth intercostal incision is relatively simple and saves operation time and the surgical incision is small, does not damage the sternum, is cosmetic, and does not cause complications such as deformed and deformed chests. Therefore, we recommend Ventricular septal defect occlusion through the left parasternal fourth intercostal incision.

## Data Availability

All data generated or analysed during this study are included in this published article.

## Abbreviations

CHD: Congenital heart disease
VSD: Ventricular septal defect;
ICU: Intensive care unit
pmVSD: Perimembranous ventricular septal defect
TEE: Transesophageal echocardiography
TTE: Transthoracic echocardiography
TTCVSD: Transthoracic closure of ventricular septal defect
TVCVSD: Transvenous closure of ventricular septal defect
RVSD: Repair of ventricular septal defect;
CPB: Cardiopulmonary bypass
MV: Mechanical ventilation
PH: Pulmonary hypertension
